# Epirubicin: a new entry in the list of fetal cardiotoxic drugs? Intrauterine death of one fetus in a twin pregnancy. Case report and review of literature

**DOI:** 10.1186/s12885-015-1976-4

**Published:** 2015-12-16

**Authors:** Marialuisa Framarino-dei-Malatesta, Giuseppina Perrone, Antonella Giancotti, Flavia Ventriglia, Martina Derme, Isabella Iannini, Valentina Tibaldi, Paola Galoppi, Paolo Sammartino, Gianluca Cascialli, Roberto Brunelli

**Affiliations:** Department of Gynecologic Obstetrics and Urology Sciences, University of Rome “Sapienza”, Rome, Italy; Department of Pediatrics, University of Rome “Sapienza”, Rome, Italy; Department of Surgery “Pietro Valdoni”, University of Rome “Sapienza”, Rome, Italy

**Keywords:** Epirubicin, Cardiotoxicity, Twin pregnancy, Fetal death, Breast cancer in pregnancy

## Abstract

**Background:**

Current knowledge indicate that epirubicin administration in late pregnancy is almost devoid of any fetal cardiotoxicity. We report a twin pregnancy complicated by breast cancer in which epirubicin administration was causatively linked to the death of one twin who was small for gestational age (SGA) and in a condition of oligohydramnios and determined the onset of a transient cardiotoxicity of the surviving fetus/newborn.

**Case presentation:**

A 38-year-old caucasic woman with a dichorionic twin pregnancy was referred to our center at 20 and 1/7 weeks for a suspected breast cancer, later confirmed by the histopathology report. At 31 and 3/7 weeks, after the second chemotherapy cycle, ultrasound examination evidenced the demise of one twin while cardiac examination revealed a monophasic diastolic ventricular filling, i.e. a diastolic dysfunction of the surviving fetus who was delivered the following day due to the occurrence of grade II placental abruption. The role of epirubicin cardiotoxicity in the death of the first twin was supported by post-mortem cardiac and placental examination and by the absence of structural or genomic abnormalities that may indicate an alternative etiology of fetal demise. The occurrence of epirubicin cardiotoxicity in the surviving newborn was confirmed by the report of high levels of troponin and transient left ventricular septal hypokinesia.

**Conclusion:**

Based on our findings we suggest that epirubicin administration in pregnancy should be preceded by the screening of some fetal conditions like SGA and oligohydramnios that may increase its cardiotoxicity and that, during treatment, the diastolic function of the fetal right ventricle should be specifically monitored by a pediatric cardiologist; also, epirubicin and desamethasone for lung maturation should not be closely administered since placental effects of glucocorticoids may increase epirubicin toxicity.

## Background

The rapidly changing sociocultural and epidemiological scene seems to increase in the near future the incidence of breast cancer in pregnancy (BCP). Recent years have witnessed a rising age at childbearing in Western countries. In Italy the mean age at first delivery increased from 29.8 years in 1995 to 31.5 years in 2013 [1]. At the same time, age at breast cancer onset in Italy has reportedly decreased, and the incidence rates for breast cancer in non-pregnant women under 45 years increased from 20.06 per 100,000 in 1980 to 32.85 per 100,000 in 2015 [2]. The increasing age at childbearing and younger age at breast cancer onset therefore imply an increased risk of BCP.

Therapeutic approaches in BCP depend on tumor stage, tumor biology, gestational age and patient’s wishes. Systemic chemotherapy may be required before or after surgery, and benefits for the mother must be compared with the potential harm to the fetus from in utero exposure to chemotherapeutics. The adhesion of patients with BCP to standard protocols based on the administration of anthracyclines/alkylating agents is highly recommended [3] as it grants patients with BCP the same disease-free interval and overall survival rates observed for non-pregnant patients with the same stage of disease [4, 5].

All chemotherapeutics are potentially teratogenic or may induce toxicity and organ dysfunction in the fetus but current knowledge indicate that anthracyclines including epirubicin administration in late pregnancy is almost devoid of any fetal cardiotoxicity.

We report a case of dichorionic pregnancy complicated by breast cancer, in which epirubicin administration was associated to the death of one twin and to the contemporary evidence of a reversible cardiotoxicity of the surviving fetus/newborn.

## Case presentation

A 38-year-old caucasic woman, G1P0, with a dichorionic twin pregnancy was referred to our center at 20 and 1/7 weeks for an excisional breast biopsy due to a suspected breast cancer. The patient underwent right external quadrantectomy with first level lymphnode (LN) dissection. Neither family history for breast cancer nor previous surgical interventions were reported. The pathology report showed an invasive and poorly differentiated (G3) ductal carcinoma not otherwise specified (NOS) measuring 2 cm in diameter (pT1). Examined LN (10) were negative for metastasis. The immunohistochemical evaluation [absent estrogen receptors (ER 0 %) and C-erb-Neu expression, positive progesteron receptors (PgR 40 %), and Ki-67 67 %] suggested a high risk of relapse, prompting the start of adjuvant chemotherapy. Maternal echocardiogram and laboratory tests were all within the normal range. A chemotherapy regimen based on epirubicin 90 mg/ m2 and cyclophosphamide 600 mg/m2 was started at a gestational age of 27 and 0/7 weeks; overall, the patient received 2 cycles of chemotherapy on a 21 days outpatient basis.

A complete assessment of fetal well-being, including the combined evaluations of fetal heart rate short term variation (STV), the largest vertical pocket of amniotic fluid (LVP-AF), pulsatility indices of the umbilical artery (UA-PI), middle cerebral artery (MCA-PI) and ductus venosus (DV-PIV), was performed before the start of chemotherapy and weekly thereafter; control of fetal growth pattern was scheduled every 3 weeks.

At baseline fetal ultrasound evaluation (26 and 6/7 weeks), one twin (A) displayed normal anatomy and was scored as small for gestational age (SGA), due to an estimated fetal weight (EFW) < 10 percentile for gestational age (679 gr) in the absence of signs of chronic placental dysfunction including fetal circulatory redistribution (UA-PI: 1 and MCA-PI: 1.5) and/or abnormal Doppler analysis of the uterine arteries (mean resistence index: 0.4); for twin A a condition of oligohydramnios was also evidenced (LVP-AF: 15 mm).

**Fig. 1:**
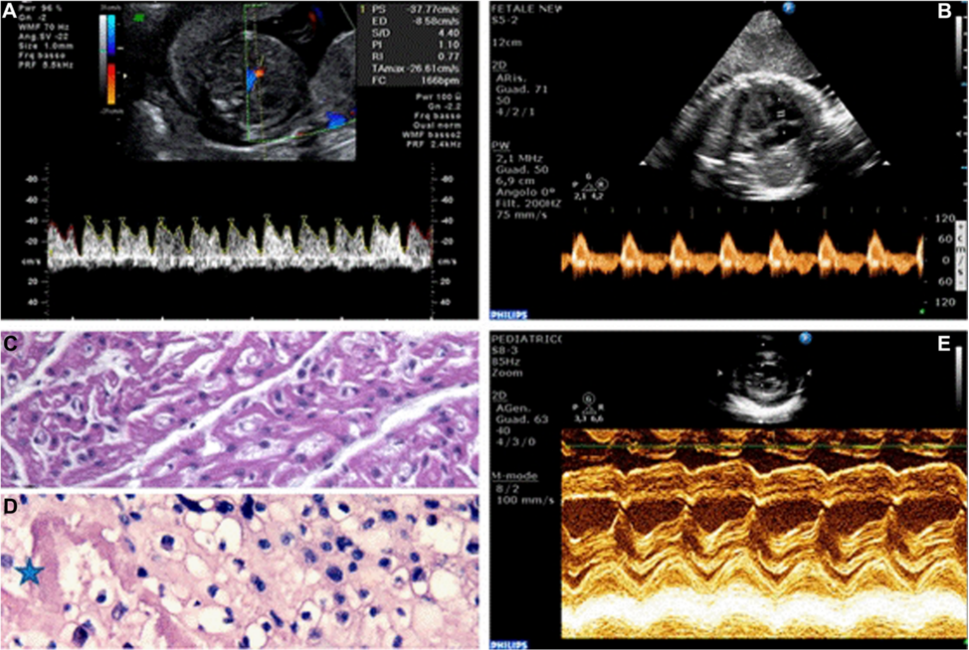
Panel **a**: Sonogram showing a significantly elevated DV-PIV in twin A. Panel **b**: Echocardiogram of twin B. Four chamber view. PW Doppler of flow through tricuspidal valve. Monofasic diastolic filling of the right ventricle; hallmark of diastolic function. Panel **c:** Myocardial severe interstitial edema with fiber dissociation and sporadic vacuolar myocyte degeneration of the twin A fetal heart. Panel **d**: Hypertrophic vacuolization and nuclear pleomorphism of extravillous throphoblast, with interstitial edema and areas of fibrinoid necrosis of placenta (✷). Panel **e**: Neonatal echocardiography of twin B. M-mode long axis of the left ventricle. Evidence of mild septal hypokinesia with an overall preserved global contractility

Fetal surveillance was completely unremarkable for both twin A and B during the two weeks that followed the first cycle of chemotherapy. At 31 and 0/7 weeks, immediately after the second chemotherapy cycle, all parameters of twin B were scored as normal. Twin A presented an unaltered growth pattern (EFW <10 percentile) and a persistent oligohydramnios (LVP-AF of 12 mm) along with normal UA-PI (1.19), MCA-PI (1.6) and STV (6.3 msec); of note, right cardiac sections appeared dilated and an abnormally high DV-PIV (1.1) was recorded, although with no evidence of reverse flow (Fig. 1 Panel a). Antenatal corticosteroids were administered (desamethasone 12 mg, 24 h apart). The following day (31 and 1/7 weeks), STV values were normal (5.9 and 6.3 msec for twins A and B, respectively).

At 31 and 3/7 weeks, ultrasounds evidenced the demise of twin A; an echocardiogram of twin B showed a monophasic diastolic filling addressing a diastolic dysfunction of the right ventricle, together with a mild hypokinesia of the ventricular septum (Fig. 1 Panel b).

At 31 and 4/7 weeks of gestation, twin B (2028 gr) was delivered by emergency cesarean section due to grade II placental abruption (Apgar score 7/9 at 1 and 5 min, respectively).

The autopsy of twin A (900 g) did not evidence structural abnormalities; cardiac histology showed severe myocardial interstitial edema with fiber dissociation and sporadic vacuolar myocyte degeneration (Fig. 1 Panel c). The only other remarkable findings were found in twin A extravillous throphoblast (hypertrophic vacuolization and nuclear pleomorphism) and chorionic villi (interstitial edema and fibrinoid necrosis) (Fig. 1 Panel D). Comparative genomic hybridization (CGH) analysis, performed on umbilical cord extracted DNA, did not show any genomic rearrangement.

Routine laboratory tests obtained for twin B during the first day of life (DOL) were normal; however, a documented significant septal hypokinesia (Fig. 1 Panel e), confirmed by a pediatric cardiologist with 20 years of experience in perinatal cardiac assessment, was associated to extremely high concentrations of troponin (Tn1) (Tn1: 0.21 μg/L; reference < 0.0014 μg/L); Tn1 was still abnormally elevated on DOL 19 (Tn1: 0.06 μg/L) but progressively decreased back to normal values on DOL 40. Pediatric cardiology examinations were regularly performed (every four weeks) up to six months of age and confirmed regular post-natal cardiac function.

Overall, the causative role of epirubicin cardiotoxicity in the death of twin A is supported by various evidences including: 1- the post-mortem cardiac examination revealing the hallmarks of subacute anthracycline toxicity, i.e. massive interstitial edema without cellular infiltrates and myofibrillar damage/vacuolization [6]; 2- the histologic findings in both chorionic villi and extravillous throphoblast, well fitting the described placental effects of anthracycline exposure [7]; 3- the manifestation of acute myocardial diastolic dysfunction, evidenced by the enlarged right cardiac chambers and an elevated ductus venosus pulsatility index, preceding fetal death; 4 – the absence of structural or genomic abnormalities that may indicate an alternative etiology of fetal demise. Of note, neither arterial Doppler nor STV evaluation anticipated the impending fetal demise.

## Conclusions

The first trimester is the most critical time regarding teratogenic effects. The blastocyst is resistant to teratogenic drugs in the first 2 weeks from conceptions whereas the administration of chemotherapy during organogenesis from 4 to 13 weeks of pregnancy is associated with an increased risk of miscarriage or congenital malformations [8] as largely documented from case reports, case series and collected reviews [9–11]. Second and third trimester chemotherapeutics exposure after the end of organogenesis, does not usually increase the teratogenic risk but may cause neurocognitive development disorders and increasing risk of intrauterine growth retardation (IUGR), pre-term labour and low birth weight [12–16].

According to SOGC guidelines, we should administer the standard regimens based on a combination of anthracyclines/alkylating agents after the end of the first trimester [17].

The first prospective collection of data addressing the issue of antracyclines safety profile in pregnancy was first reported by the Texas MD Anderson Cancer Center back in 1999. These authors treated 24 pregnant patients with primary or recurrent cancer of the breast managed with a standardized protocol of 5-fluorouracil + doxorubicin + cyclophosphamide (FAC) chemotherapy in the second and third trimester of pregnancy and did not report an increased rate of congenital anomalies [18]. In the same year, a French survey reporting 12 patients with BCP alternatively treated with FAC, 5-fluorouracil + epirubicin + cyclophosphamide (FEC), epirubicin + cyclophosphamide (EC) or doxorubicin + cyclophosphamide (AC) evidenced only one case of intrauterine death at 30 weeks of gestation [19]. In a retrospective, cohort study evaluating the fetal risks involved in the administration of cancer chemotherapy during gestation, one fetus died after second trimester exposure to epirubicin, vincristine and prednisone but no malformation was detected [20]. Hahn et al. extended the previous evidences from the MD Anderson Cancer Center and confirmed the absence of congenital birth defects in fetuses exposed to anthracyclines chemotherapy in utero; indeed, only three children reported congenital malformations in a group of 57 women treated up to 2006, with a median number of four FAC cycles given during pregnancy; one neonate was born with Down syndrome, one with ureteral reflux, and a third with club foot [21]. Ring et al. evaluated 16 out of 28 BCP patients receiving anthracyclines-based chemotherapy during pregnancy without reporting any congenital birth defect [22].

The German Breast Group issued the first International Recommendations on BCP and confirmed the safety of anthracyclines [23]. An international consensus meeting held in 2010 confirmed that anthracyclines can be used in the setting of BCP [24] and RCOG guidelines assigned an Evidence level 3 to the statement that anthracycline-based chemotherapy in the second and third trimesters can be administered with minimal risk to the developing fetus [25].

Anthacyclines display well-known cardiotoxic effects: age, cumulative dose and previous radiotherapy increases the rates of cardiac damage in children and adults [26]. The molecular mechanisms underlying antracyclines cardiotoxicity are not fully understood, but include alterations of cell membranes fluidity and ion transport with generation of reactive oxygen species by iron-anthracycline complexes, leading to lipid peroxidation and membrane damage [27] and the impairment of DNA repair through the interaction with the topoisomerase-II-beta enzyme in myocytes [28]. Increasing evidences show that the extracellular matrix plays a complex and diverse role in some processes initiated by anthracyclines that finally lead to cardiac damage [29]. Notably, fetal myocardium is theoretically more vulnerable to damage by chemotherapeutics because fetal myocytes are smaller than adult ones, and contain fewer sarcomeres and mitochondria [30].

Fetal safety during the administration of anthracycline-based chemotherapy in pregnancy is of theoretical concern because anthracyclines can cross the placenta, even if their fetal plasma concentrations are lower than those found in the mother, and have cumulative toxicity [31]. Available data provide only limited experimental and clinical data on the transplacental transfer of these chemotherapeutics in pregnant women; in a baboon model, fetal plasma concentrations of doxorubicin, epirubicin and paclitaxel were about 7.5 %, 4.0 %, and 1.4 %, of the respective maternal concentrations [32]. Fetal blood samples from pregnant rats receiving doxorubicin showed a plasmatic concentration that was 6.2 % that of the mother; interestingly, neither Doppler analysis nor heart microstructure or cellular DNA turnover and apoptosis were influenced by doxorubicin exposure [33].

Owing to the molecular weight of doxorubicin is 580 dalton, there is an incomplete transfer of the drug across the placental barrier [8]. However, the transplacental passage cannot be simply predicted from the physical-chemical properties of the drugs like the molecular weight. Really, while assessing fetal plasma drug concentrations, the functional expression of many members of the ATP-binding cassette (ABC) efflux transporters that are highly expressed in the human placenta, should be adequately considered; indeed, these transporters prevent the trans-placental transfer of cytotoxic compounds present in the maternal circulation, therefore protecting the fetus [34, 35]; specifically, anthracyclines and taxanes are substrates for ABC-transporters like the major placental drug-transporting P-glycoprotein, that keeps low the fetal plasma concentrations of these harmful compounds [36].

Anthracyclines do not collectively share the same low rate of transplacental transfer. Indeed, idarubicin, being more lipophilic than other antracyclines, easily crosses the placenta; Germann reported one fetal death and one case of reversible heart dysfunction in a group of patients affected by acute myeloid leukemia receiving idarubicin-based chemotherapy during the third trimester of pregnancy [37]. Similarly, Baumgartner reported one case of reversible fetal cardiomyopathy following the use of idarubicin during pregnancy [38] while the occurrence of a severe idarubicin-related cardiotoxicity in a newborn was described in a swiss study [39]. Altogether, these findings suggest a close fetal monitoring during idarubicin based chemotherapy; long-term outcomes of idarubicin exposed children need further investigations.

Unlike idarubicin, doxorubicin and epirubicin, due to their low levels in fetal plasma, may be administered during the second and third trimesters without significant risk of fetal myocardial dysfunction. At first, clinicians gained some experience on the safety profile of these two antracyclines from case reports and small case series [40]. Further reassuring evidence was granted by an Italian review reporting that only 13/out of 403 (3 %) children exposed to these anthracyclines during late pregnancy developed short-term cardiac complications [41]. Azim et al. reported that different epirubicin and doxorubicin regimens administered in adjuvant, neoadjuvant and metastatic settings (23 patients and 3 patients, respectively) did not adversely affect the course of pregnancy or fetal/neonatal outcome [42]. In a small cohort of patients, even a dose-dense antracyclines chemotherapy administered every two weeks did not involve a higher risk of fetal complications [43].

In a prospective case–control clinical study, Gziri found that maternal and fetal cardiac functions were not significantly hampered by anthracyclines exposure in pregnancy but rather displayed only minor changes of the myocardial performance index and the tricuspid inflow devoid of any clinical relevance [44].

Notably, epirubicin in pregnancy has a shorter terminal half-life than doxorubicin due to its combined glucuronization by the liver and the placenta [45] and therefore displays a better therapeutic index with fewer systemic and cardiotoxic effects. In an Austrian study, all three patients managed at the University Hospital of Vienna with six courses of FEC neoadjuvant chemotherapy delivered healthy newborns [46]. Others case reports on multidrugs regimens including epirubicin as adjuvant treatment for pregnant women with high-risk breast cancer failed to show any fetal cardiotoxicity [47, 48].

Some authors report that weekly epirubicin schedule seems particularly safe because it decreases the potential adverse events and simultaneously facilitates a close monitoring of pregnancy [49].

Overall, anthracyclines emerge as theoretically safe during the late trimesters of pregnancy, fetal concentrations being 100/1000-fold lower than adults as a result of the high molecular weight, the hydrophilic charge leading to a limited transplacental passage and the active clearance operated by the placental P-glycoprotein transporter. Indeed, despite the difficulty of comparing different agents and schedules used for BCP, fetal cardiotoxicity never emerged as a major problem of anthracyclines administration; in particular the available evidences indicate that epirubicin harmful effects on fetal heart are very limited with only one reported case of transient ventricular hypokinesia [41].

In this otherwise quite reassuring scenario, we provide evidence that, in a twin pregnancy complicated by breast cancer, epirubicin administration was causatively linked to the death of one twin and to the onset of a reversible cardiotoxicity of the surviving fetus/newborn.

The ultimate cause of twin A great susceptibility to the cardiotoxic action of epirubicin remains elusive. Anthracyclines are concentrated up to nine times more in the amniotic fluid than in fetal plasma [32]; in this regard, the presence of oligohydramnios and the histologic evidences of altered extravillous throphoblast and chorionic villi, suggest the a putative contribution of an abnormal amniotic fluid dynamics to the increased/prolonged toxicity of epirubicin in twin A. Further support to the hypothesis of an hampered epirubicin farmacokynetics is offered by the circumstantial evidences that the succumbing SGA twin A died shortly after the administration for lung maturation of glucocorticoids; these steroids, among a myriad of actions, are known to downregulate the throphoblast expression of the detoxifying P-glycoprotein transporter [50]. Epirubicin cardiotoxicity was also evident, although to a lesser extent, in Twin B as shown by 1- the prenatal findings of an isolated mild right ventricular diastolic dysfunction (reflecting the greater after load of this ventricle in the fetal circulation) and 2- postnatal recording of increased Tn1, associated to a transient left ventricular septal hypokinesia [51].

In conclusion, with reference to the above mentioned considerations, we suggest that a precautional use of epirubicin in pregnancy should include; 1- the screening of oligohydramnios since this condition may putatively increase epirubicin cardiotoxicity 2- a timely surveillance by a pediatric cardiologist of the diastolic function of the fetal right ventricle, because the other indices of fetal well being are poorly predictive of an impending fetal cardiac decompensation 3- the avoidance of a close administration of epirubicin and desamethasone since glucocorticoids may hamper placental metabolism of epirubicin, ultimately increasing its toxicity.

## Consent to publish

Written informed consent was obtained from the patient for publication of this Case report and any accompanying images. A copy of the written consent is available for review by the Editor of this journal.
